# Supplementary figures and images for: LDOC1 Suppresses Microbe-Induced Production of IL-1β in Human Normal and Cancerous Oral Cells through the PI3K/Akt/GSK-3β Axis
Source: Cancers (Basel). 2020 Oct 27;12(11):3148. doi: 10.3390/cancers12113148 (PMC7694066; doi:10.3390/cancers12113148)

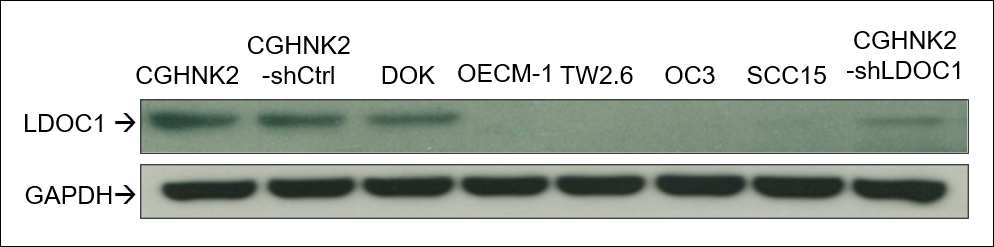

Supplement: Supplementary file 1 [file cancers-12-03148-s001.zip › SF1.tif]

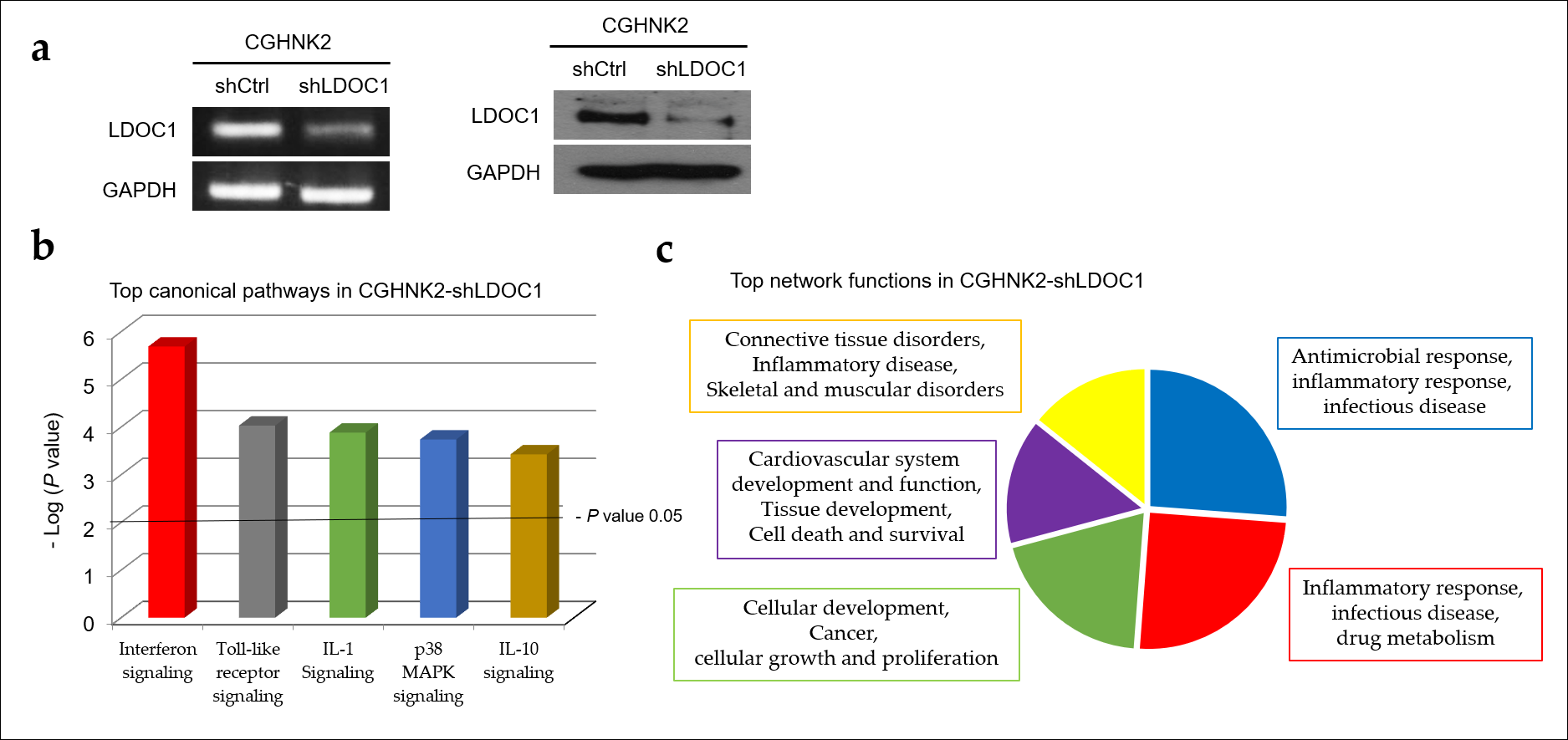

Supplement: Supplementary file 1 [file cancers-12-03148-s001.zip › SF2.tif]
